# Comparison of two different coagulation algorithms on the use of allogenic blood products and coagulation factors in severely injured trauma patients: a retrospective, multicentre, observational study

**DOI:** 10.1186/s13049-017-0463-0

**Published:** 2018-01-08

**Authors:** Alexander Kaserer, Mattias Casutt, Kai Sprengel, Burkhardt Seifert, Donat R. Spahn, Philipp Stein

**Affiliations:** 1 Institute of Anaesthesiology, University and University Hospital Zürich, Raemistrasse 100, 8091 Zürich, Switzerland; 2 Department of Anaesthesiology and Intensive Care, Cantonal Hospital Lucerne, Spitalstrasse 16, 6000 Luzern, Switzerland; 3 Department of Traumatology, University and University Hospital Zürich, Raemistrasse 100, 8091 Zürich, Switzerland; 4 Epidemiology, Biostatistics and Prevention Institute, Department of Biostatistics, University of Zurich, Hirschengraben 84, 8001 Zurich, Switzerland

**Keywords:** Coagulation algorithm, Coagulation management, Point of care measurements, Transfusion, Trauma

## Abstract

**Background:**

At the University Hospital Zurich (USZ) and the Cantonal Hospital of Lucerne (LUKS) an individualized goal-directed coagulation and transfusion algorithm was introduced and implemented before 2012 (Coagulation algorithm of the USZ: USZ-Alg; of the LUKS: LUKS-Alg). Main differences between both algorithms are: 1) A target haematocrit-range of 0.21–0.24 (USZ-Alg) vs. a lower haematocrit limit only (LUKS-Alg). 2) Blind coagulation-package in selected cases (LUKS-Alg only). 3) Factor XIII substitution is considered earlier according to the USZ-Alg.

The Aim of this study was to analyse the impact of two different coagulation algorithms on the administration of allogeneic blood products, coagulation factors, the frequency of point of care measurements and haemoglobin level during resuscitation in trauma patients.

**Methods:**

This retrospective, multicentre, observational study included all adult trauma patients with an injury severity score (ISS) ≥ 16 primarily admitted to the USZ or the LUKS in the period of 2012 to 2014. Referred patients and patients with missing/incomplete records of the initial treatment at the emergency department (ED) were excluded. Two propensity score matched groups were created using a non-parsimonious logistic regression to account for potential differences in patient and trauma epidemiology.

**Results:**

A total of 632 patients meeting the inclusion criteria were admitted to the two hospitals: 428 to the USZ and 204 to the LUKS. Two Propensity score matched groups (*n* = 172 per group) were created. Treatment with USZ-Alg compared with LUKS-Alg resulted in a lower number of patients receiving RBC transfusion (11.6% vs. 29.7%, OR 3.2, 95% CI 1.8–5.7, *p* < 0.001) and lower amount of RBC transfusion (0.5 SD 1.9 vs. 1.5 SD 3.9, *p* < 0.001). The different treatment algorithms resulted in lower mean haemoglobin levels in the USZ during resuscitation (8.0 SD 1.7 vs. 9.4 SD 1.8 g/dl, *p* < 0.001) and at admission to the ICU (8.3 SD 1.2 vs. 10.6 SD 1.9 g/dl, *p* < 0.001. Blood gas analyses to monitor treatment and haematocrit were made more frequently in the USZ (1.4 SD 0.8 vs. 1.0 SD 0.7 measurements per hour, *p* = 0.004).

**Conclusion:**

A goal-directed coagulation algorithm including a target haematocrit-range including frequent and repeated haematocrit measurement may lead to less transfusion of RBC compared to only a lower haematocrit limit, when treating severely traumatized patients.

**Electronic supplementary material:**

The online version of this article (10.1186/s13049-017-0463-0) contains supplementary material, which is available to authorized users.

## Background

Individualized goal-directed point-of-care and factor concentrate based coagulation and transfusion algorithms to treat massively bleeding patients following severe trauma have been published [1–4]. Such algorithms require adequate, fast and goal-directed replacement of plasma components and coagulation factors to tackle trauma induced coagulopathy and its consequences, particularly mortality [5, 6]. It could already be shown that the implementation of transfusion and coagulation algorithms leads to an improved outcome with a significant reduction of mortality, massive transfusion and the use of allogeneic blood products [1, 3, 7]. Coagulation management according to individualized goal-directed coagulation and transfusion algorithms is one of the key elements in the European Trauma Treatment Guidelines [8].

In 2008, the University Hospital of Zurich (USZ), Switzerland, implemented an algorithm for goal directed transfusion and coagulation management based on the pathophysiology of a developing coagulopathy in massively bleeding patients [9]. The algorithm was subsequently revised and then fully implemented in 2012 [1, 10, 11]. A similar algorithm was implemented in the Cantonal Hospital of Lucerne (LUKS), Switzerland, in 2011.

Although both algorithm base on the same principles and are largely comparable, some differences exist. These differences may have significant influence on the administration of allogeneic blood products and coagulation factors. To the best of our knowledge no study has been conducted and published comparing two goal-directed, point of care and factor based coagulation algorithms. The aim of this study was to analyse the impact of the differences between two distinct transfusion and coagulation algorithms on the administration of allogeneic blood products, coagulation factors, the frequency of point of care measurements and haemoglobin levels during resuscitation.

## Methods

This study was approved by the local ethics committee (Kantonale Ethikkommission Zürich, Switzerland, KEK ZH 2015–0309) and adheres to the Strengthening the Reporting of Observational Studies in Epidemiology (STROBE) recommendations for cohort studies [12].

### Study design and participants

This retrospective, multicentre, observational study includes all adult trauma patients (≥ 16 years) with an Injury Severity Score (ISS) ≥ 16 primarily admitted to the USZ or the LUKS between January 1, 2012 and December 31, 2014.

**Fig. 1:**
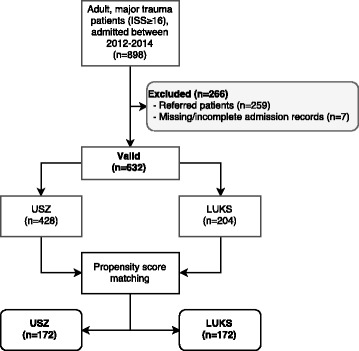
Flowchart of patient selection according to defined inclusion and exclusion criteria. LUKS: Cantonal Hospital Lucerne; USZ: University Hospital Zurich; ISS: Injury severity score

Exclusion criteria were secondary transfer to the USZ or LUKS, Age < 16 years and missing/incomplete records of the initial treatment at the emergency department (Fig. 1).

### Setting

The USZ and LUKS are two of twelve level 1 trauma centres in Switzerland. All severely injured patients are transferred by ambulance or helicopter to one of these level 1 trauma centres. A standardized clinical approach is provided in the emergency department (ED) of the USZ and LUKS consisting of a primary survey and further treatment according to ATLS® or ETC®. The trauma staff at the ED contains at least one senior and one junior anaesthetist one senior and one junior trauma surgeon. An initial whole-body CT scan is performed in all major trauma patients as soon as possible to evaluate their relevant injuries and to determine further treatment [13].

### Transfusion and coagulation algorithm

At the USZ in 2008 an individualized goal-directed transfusion and coagulation algorithm was introduced, revised and fully implemented until 2012 (USZ-Alg) [9–11]. At the LUKS, a similar coagulation algorithm was introduced and implemented during 2011 (LUKS-Alg)*.* Both algorithm guide the transfusion and coagulation management for all massively bleeding trauma patients.

#### Basics of both algorithm

On admission to the ED, blood samples to determine haemoglobin level, platelet count, fibrinogen level, factor V and XIII activity, international normalized ratio (INR), aPTT, blood gas analysis (ABGA) and ROTEM® (Rotational thromboelastometry, TEM International, Munich, Germany) analysis are taken. Haematology assays and factor activity measurement are available 24 h a day, within 30 min. ROTEM® measurements include EXTEM (tissue factor activated extrinsic pathway), INTEM (ellagic acid activated intrinsic pathway), FIBTEM (containing platelet inhibitor cytochalasin D, evaluating the contribution of fibrinogen to clot formation) and APTEM (containing aprotinin to inhibit plasmin to evaluate fibrinolysis). The first step is to screen patient’s medical history and medication to identify factors affecting coagulation (antiplatelet drugs, heparin, oral anticoagulants or history of immune reactions altering coagulation). General target values include normothermia, normocalcemia, normal acid-base status, adequate haematocrit, permissive hypotension (mean arterial pressure 55–60 mmHg). Fluid resuscitation should primarily be performed with balanced crystalloid solutions. If colloids are considered to be useful, only gelatin administration is recommended in both algorithms. Tranexamic acid (TXA), fibrinogen concentrate, 4 factor prothrombin complex concentrate (PCC), factor XIII and allogeneic blood products are administered according to laboratory, ABGA and ROTEM® findings. If target values are reached, diffuse bleeding continues and a DIC is not likely, recombinant factor VIIa may be evaluated.

#### Main differences between both algorithm

- For the RBC transfusion management, a target haematocrit-range of 0.21–0.24 is specified in USZ-Alg, whereas in LUKS-Alg only a lower haematocrit limit of 0.21 is defined.
- LUKS-Alg contains the option for selected Trauma patients (age < 50 years, no fatal injury, temperature > 35 °C and diffuse bleeding) to administer the following transfusion - and coagulation package blindly and not goal-directed: 2 g Tranexamic acid, 4 g Fibrinogen, 1′000 U Prothrombin complex concentrate, 4 RBCs (blood group 0 negative) and 1250 U factor XIII.
- In the LUKS-Alg, Factor XIII 1250 U (15 U/kg) is considered if FIBTEM maximum clot firmness stays ≤ 7 mm despite fibrinogen administration, or factor XIII activity is < 60%. According to the USZ-Alg Factor XIII may be administering blindly after 6 g of fibrinogen or at factor XIII activity < 60% to improve clot stabilization.

The LUKS-Alg is depicted in detail in the supplemental material (Additional file 1). The USZ-Alg is depicted in detail in the publication of Theusinger et al. [10].

### Variables and data collection

Patient demographics, laboratory values at admission (samples collected within the first minutes after admission to the hospital) and injury epidemiology were extracted from the internal database which is collected and entered by professional medical coders responsible for the data acquisition to the TraumaRegister DGU(R) of the German Trauma Society and copied to a designated spreadsheet (Microsoft® Office Excel 2010, Microsoft® Corporation, Redmond, USA). Additionally, the following variables concerning initial resuscitation in the emergency department (hospital admission to ICU admission) were extracted from the anaesthesia records: use of resuscitation fluids, factor concentrates (PCC, factor XIII, recombinant factor VIIa), TXA, vasopressor use (yes/no), duration of treatment and the use of allogeneic blood products such as RBC, FFP and PLT. Multiple cross checks have been performed to ensure high data quality. The median proportion of missing values was 1.4%, IQR 0 to 4.7%.

### Endpoints and outcome variables

The primary endpoints were the comparison between centres / algorithms in the use of allogenic blood products and coagulation factors. Secondary endpoints were the frequency of point of care measurements and haemoglobin level during resuscitation and at admission ICU.

### Statistical analyses

Numerical data reported as mean and standard deviation (SD) or median [Q1;Q3] in the case of skew data distribution and ordinal data (GCS). Categorical data shown in absolute numbers (n) and percent (%). For the primary analysis, two propensity score matched groups (*n* = 172 per group) were computed using a non-parsimonious logistic regression. Skew data was logarithmically transformed (time in the ED, time prehospital, platelet count, lactate). Propensity score matching was performed on the logit scale with a caliper of 0.2 standard deviations of the logit of the propensity score. Missing values were replaced by multiple regression imputation for the respective analysis (dataset was generated using 10 iterations of automatic (linear or logistic) regressions depending on the type of the variable). The model fit was assessed using the Hosmer–Lemeshow test. The model was well calibrated (chi-square with 8 degrees of freedom = 11.4, *p* = 0.16). Standardized difference was calculated for the (partially logarithmically transformed) explanatory variables (Table 1) to assess the balance on base line characteristics after propensity score matching. Odds ratios (OR) and 95% CI were calculated using logistic regression. The Mann-Whitney test was used to compare continuous data between the groups. Statistical significance was set as a two-tailed *p*-value ≤ 0.05. All statistical analyses were performed by IBM SPSS Statistics 22 (Armonk, NY: IBM Corp).

**Table 1:** Overview of patient characteristics, mechanisms of injury, management and first laboratory values determined at admission in the propensity score matched groups comparing two 1 trauma centres (LUKS: Cantonal Hospital Lucerne, USZ: University Hospital Zurich)

	USZ (*n* = 172)	LUKS (*n* = 172)	Standardized difference
Age (years)	53.6 (21.8)	51.1 (19.7)	0.09
Sex (male)	119 (69%)	121 (70%)	0.03
Heart rate (bpm) on admission	90 (23) (*n* = 169)	91 (21) (*n* = 167)	0.04
Systolic blood pressure (mmHg) on admission	131 (27) (*n* = 171)	132 (31) (*n* = 168)	0.04
Penetrating trauma	8 (5%)	6 (4%) (n = 169)	0.06
GCS on scene	13 [6;15] (*n* = 167)	13 [6;15] (*n* = 169)	0.09
ISS	27.8 (14.5)	27.9 (11.5)	0.01
Time (min.) prehospital	65 [55;84]	70 [55;90] (*n* = 143)	0.01^#^
Time (min.) ED	145 [100;255]	170 [105;309] (n = 143)	0.11^#^
Prehospital intubation	48 (28%)	56 (33%)	0.10
Vasopressor use (in the ED)	90 (52%)	93 (54%)	0.03
First laboratory values determined after admission to the ED			
Haemoglobin (g/dl)	12.1 (2.1) (*n* = 166)	12.1 (2.3) (*n* = 169)	0.00
Platelet count (G/l)	186 [152;225] (*n* = 167)	197 [164;227] (*n* = 164)	0.10^#^
Base Excess (mmol/l)	−3.5 (4.9) (*n* = 165)	−2.8 (5.5) (*n* = 165)	0.12
Lactate (mmol/l)	1.7 [1.1;2.6] (*n* = 166)	1.7 [1.0;3.0] (*n* = 148)	0.13^#^
Quick’s value (%)	71 (22) (*n* = 167)	73 (19) (*n* = 166)	0.09
Fibrinogen (g/l)	2.3 (1.0) (*n* = 160)	2.3 (0.8) (*n* = 162)	0.07

Data reported as frequency (n) with percentage (%), mean (SD) or median [Q1;Q3]. Standardized difference for the explanatory variables = absolute difference in means or proportions divided by pooled standard deviation. #: standardized difference computed for logarithmically transformed variable. Standardized difference is ≤ 0.13 for all explanatory variables, stating an acceptable balance between the matched groups

*BPM* beats per minute, *ED* emergency department, *GCS* glasgow coma scale, *ISS* injury severity score

## Results

A total of 632 patients meeting the inclusion criteria were admitted to the two hospitals: 428 patients were admitted to the USZ and 204 to the LUKS (Fig. 1). Two Propensity score matched groups (*n* = 172 per group) were created with the explanatory variables in Table 1. The absolute standardized difference after matching was ≤0.13 for all explanatory variables, stating an acceptable balance between the matched groups (Table 1).

### Usage of blood products, coagulations factors and resuscitation fluids

**Fig. 2:**
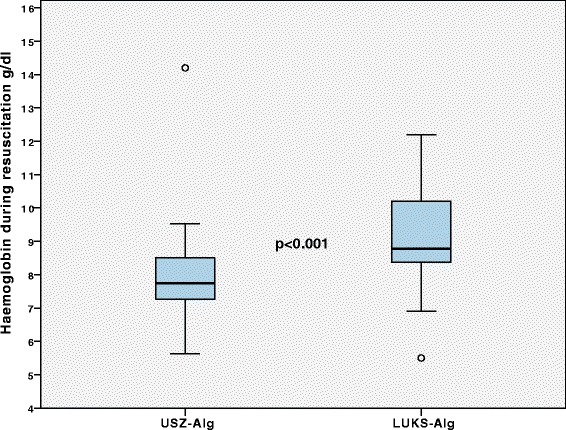
Mean haemoglobin during resuscitation at the ED. In Patients from the propensity score matched groups requiring at least 1 RBC, mean haemoglobin level during resuscitation at the emergency department was significantly different between both trauma centres: *p* < 0.001. RBC: *red* blood cell concentrate; ED: emergency department; USZ-Alg: coagulation and transfusion algorithm of the University Hospital Zurich; LUKS-Alg: coagulation and transfusion algorithm of the Cantonal Hospital Lucerne

**Fig. 3:**
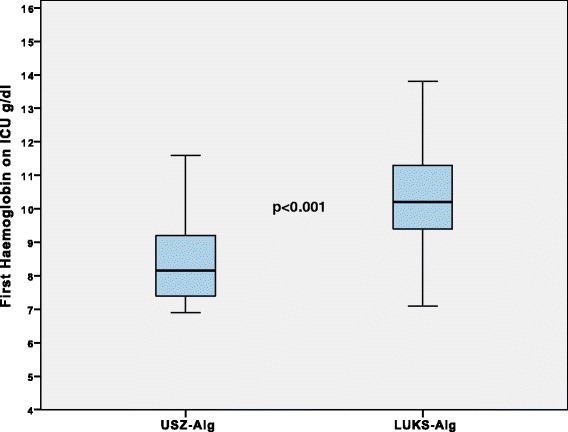
First haemoglobin on ICU. In patients from the propensity score matched groups requiring at least 1 RBC, first haemoglobin level on admission to the ICU was significantly different between both trauma centres: *p* < 0.001. ICU: intensive care unit; RBC: *red* blood cell concentrate; USZ-Alg: coagulation and transfusion algorithm of the University Hospital Zurich; LUKS-Alg: coagulation and transfusion algorithm of the Cantonal Hospital Lucerne

Treatment with USZ-Alg compared with LUKS-Alg resulted in a lower number of patients receiving RBC transfusion (11.6% vs. 29.7%, OR 3.2, 95 CI 1.8–5.7, *p* < 0.001, Table 2) and a lower amount of RBC transfusion (0.5 SD 1.9 vs. 1.5 SD 3.9, *p* < 0.001, Table 3). Concerning FFP and PLT transfusions, the number of patients transfused and the amount of transfused units did not differ between the algorithms. In patients treated according to the USZ-Alg, mean haemoglobin level in transfused patients was lower during resuscitation (8.0 SD 1.7 vs. 9.4 SD 1.8 g/dl, *p* < 0.001) and at ICU admission (8.3 SD 1.2 vs. 10.6 SD 1.9 g/dl, *p* < 0.001, Figs. 2 and 3).

**Table 2:** Differences in the number of patients receiving allogeneic blood products, coagulation factors and resuscitation fluids (independent of amount) between the propensity score matched groups (LUKS: Cantonal Hospital Lucerne, USZ: University Hospital Zurich)

	USZ (*n* = 172)	LUKS (*n* = 172)	OR (95% CI)	*p*-value
RBC	20 (11.6%)	51 (29.7%)	3.2 (1.8–5.7)	< 0.001
FFP	4 (2.3%)	9 (5.2%)	2.3 (0.7–7.7)	0.16
PLT	10 (5.8%)	6 (3.5%)	0.6 (0.2–1.6)	0.31
TXA	76 (44.2%)	66 (28.6%)	0.8 (0.5–1.2)	0.29
Fibrinogen	42 (24.4%)	59 (34.3%)	1.6 (1.0–2.6)	0.04
PCC	17 (9.9%)	41 (23.8%)	2.9 (1.5–5.3)	0.001
Factor XIII	15 (8.7%)	6 (3.5%)	0.4 (0.1–1.0)	0.04
Starch	5 (2.9%)	37 (21.5%)	9.2 (3.5–23.9)	< 0.001
Gelatin	47 (27.3%)	27 (15.7%)	0.5 (0.3–0.8)	0.009

Data reported as frequency (n) with percentage (%). Odds ratio (OR) with 95% confidence intervals (CI) and *p*-values were calculated using logistic regression. Level of significance 0.05

*FFP* fresh frozen plasma, *FXIII* coagulation factor XIII, *PCC* 4 factor prothrombin complex concentrate, *PLT* platelet concentrate, *RBC* red blood cell concentrate, *TXA* tranexamic acid

**Table 3:** Differences in the amount/quantity of administered allogeneic blood products, coagulation factors and resuscitation fluids between the propensity score matched groups (LUKS: Cantonal Hospital Lucerne, USZ: University Hospital Zurich)

	USZ (*n* = 172)	LUKS (*n* = 172)	*p*-value
RBC (U)	0.5 (1.9)	1.5 (3.9)	< 0.001
FFP (U)	0.1 (0.6)	0.4 (2.2)	0.15
PLT (U)	0.1 (0.3)	0.1 (0.4)	0.32
FFP:RBC (ratio)	0.1 (0.3)	0.1 (0.3)	0.91
Fibrinogen (g)	1.1 (2.6)	1.5 (3.0)	0.05
PCC (IU)	124 (408)	454 (1011)	< 0.001
Factor XIII (IU)	116 (388)	51 (282)	0.04
Crystalloid (ml)	2130 (2642)	3944 (4064)	< 0.001
Starch (ml)	13 (94)	146 (319)	< 0.001
Gelatin (ml)	347 (722)	142 (392)	0.004

Data reported as mean (SD). The *p*-value (Mann-Whitney test) was calculated between the groups. Level of significance 0.05

*FFP* fresh frozen plasma, *FXIII* coagulation factor XIII, *PCC* 4 factor prothrombin complex concentrate, *PLT* platelet concentrate, *RBC* red blood cell concentrate

If treated according to the USZ-Alg, significantly more patients received factor XIII, but less patients received PCC and fibrinogen concentrate (Table 2). The mean overall dose was higher for factor XIII and lower for PCC and fibrinogen if USZ-Alg was applied (Table 3). None of the patients received recombinant factor VIIa. Patients treated according to the USZ algorithm received significantly lower volumes of crystalloids (Table 3), less frequently and lower volumes of hydroxyethyl starch but more frequently and higher volumes of gelatin (Tables 2 and 3).

### Point of care measurements during resuscitation

In patients receiving RBC, more blood gas analyses were made at the USZ (1.4 SD 0.8 vs. 1.0 SD 0.7 measurements per hour, *p* = 0.004). No significant differences in the amount of ROTEM® analyses could be observed between both algorithms (0.5 SD 0.4 vs. 0.5 SD 0.4 measurements per hour, *p* = 0.83).

## Discussion

The treatment of severely injured patients with individualized goal-directed factor based coagulation and transfusion algorithms is becoming more and more common nowadays and many hospitals already implemented such algorithms. Although common practice, to the best of our knowledge this is the first study investigating the impact of differences in coagulation algorithms on the administration of allogeneic blood products and coagulation factors, point of care measurements and haemoglobin level during resuscitation and at admission ICU.

Our main findings were 1) a target haematocrit-range may lead to less transfusion of RBC compared to a lower haematocrit limit only, 2) especially in patients requiring allogenic blood products frequently repeated haemoglobin measurement may be crucial to monitor transfusion management.

Following to the USZ-Alg, frequency and amount of RBC transfusions were significantly lower. In severely injured trauma patients RBC transfusion is only recommended according to evidence based low transfusion triggers [14–17]. As RBC do not contain any coagulation factors RBC transfusions lead to dilution of coagulation factors, which may worsen the bleeding [9]. Therefore, in the USZ-Alg a haematocrit-range as RBC transfusion target is established, avoiding excessive RBC transfusion. This may additionally be reflected by the higher amount of ABGA performed per hour at the USZ to focus on the haematocrit-range in transfused patients compared to the LUKS, where the haemoglobin level is considered to be kept upper a lower haematocrit limit only.

In Switzerland, prehospital tranexamic acid administration was initiated after the CRASH-2 trial was published in 2011 [18]. Therefore, the use of tranexamic acid may be underestimated for both centres, because prehospital administration of tranexamic acid was not analysed. No additional tranexamic acid was given and documented in the ED, if prehospital TXA was already applied. Early empirical administration of tranexamic acid is recommended by the European Trauma Treatment Guidelines to bleeding trauma patients or patients at risk of significant haemorrhage [8].

A difference in fibrinogen concentrate administration was observed, which may be due to goal-directed use according to the USZ-Alg, compared to empirical (partially) use of fibrinogen concentrate according to the LUKS-Alg. As fibrinogen concentrate substitution reduces the need for FFP and RBC transfusion in trauma patients [19], rapid assessment and correction of the initial fibrinogen level is recommended by both algorithms.

At the LUKS patients received PCC more frequently and at a higher dosage. Although PCC is part of the “blind package” for selected trauma cases according to LUKS-Alg, the European Trauma Treatment Guidelines recommend PCC administration only to bleeding patients with documented evidence of delayed coagulation initiation (goal-directed approach) and to trauma patients who are anticoagulated with vitamin K antagonists [8]. Treating patients blindly with PCC is therefore not recommended and may explain the observed difference for PCC between both centres.

The majority of trauma patients in both centres is no longer exposed to FFP transfusions. As FFP was the only source for coagulation factor XIII, this factor may reach critically low values in bleeding patients with progressive haemodilution. Therefore, measurement and early administration of factor XIII is recommended. It was shown that factor XIII stabilizes the fibrin clot in vitro [20] and reduces postoperative bleeding in cardiac surgery [21]. More coagulation factor XIII was administered at the USZ as it is earlier recommended according to the USZ-Alg.

In both trauma centres, crystalloid solutions are used primarily for fluid resuscitation. Patients at the USZ received a more restrictive volume resuscitation. Restrictive fluid resuscitation is recommended to avoid dilution of coagulation factors, which impairs coagulation additionally until the bleeding is surgically controlled [8]. On the other hand the survival > = 72 h in severely injured patient is associated with a higher amount of especially saline solutions indicating that some patients may benefit from a more generous fluid resuscitation [22]. The overall numbers of patients that received colloids were comparable between the LUKS and the USZ. Whereas at the USZ used predominantly gelatin, the LUKS used gelatin and hydroxyethyl starch equally frequently throughout the study period, despite the fact both algorithms recommend the use of gelatin. This might be explained by the fact, that the negative, non-reversible effect of starch on coagulation was postulated during the observed study period. It was shown that colloid solutions are affecting coagulation as well as they are an independent predictor of SIRS and Sepsis in severely injured patients [23–25]. Thereby especially starch worsens blood coagulation by impairing fibrin polymerization and platelet function [20, 26]. In contrast to gelatin, this negative impact on coagulation of hydroxyethyl starch is not reversible by adding coagulation factor concentrates [23, 26].

Individualized goal-directed factor concentrate-based algorithms are based on point of care measurements and routine laboratory measurements. Point of care measurements, including viscoelastic testing of the developing clot (e.g. ROTEM®) and ABGA, allow rapid and tailored coagulation and transfusion treatment [3, 27]. The FIBTEM test in viscoelastic testing with ROTEM® allows to identify low functional fibrinogen levels within 5–10 min [27]. Low levels are shown to be predictive for massive transfusion in trauma patients [19]. A ROTEM®-guided haemostatic therapy with fibrinogen concentrate and PCC led to a reduction in the administration of allogeneic blood products in trauma patients [3, 4, 28, 29]. Moreover, a TEG®-guided protocol was shown to be superior to a massive transfusion protocol with a fixed ratio strategy in patients with penetrating trauma [2]. It is therefore crucial that point of care measurements are frequently repeated to guide and monitor the coagulation and transfusion management, especially after administering coagulation factors and allogeneic blood products.

### Limitations

Our study has several limitations hat may have interfered with our results. Data analyses were performed retrospectively. However, data quality is high, as all the emergency department data have been collected independently and cross-checked. Moreover, documentation was performed according to the Good Clinical Practice guidelines. Both trauma centres have a standardized approach for the initial treatment of severely injured patients based on to leading trauma guidelines, such as ATLS® or ETC®. Nevertheless, apart from the differences in the coagulation algorithms, also other differences in the treatment of trauma patients may not be accounted for despite the fact that after matching, demographic and trauma epidemiology including admission laboratory values did not differ between the groups. From this study design, therefore only associations but no causal relationships may be derived. Treatment differences may not be limited to the investigated algorithms only, but also to undetermined differences between the study centres. Conducting a prospective multicentre study to investigate 2 coagulation algorithms in parallel would be difficult to perform, as for example blinding is not possible. Therefore, comparing two coagulation algorithms retrospectively and showing differences in the transfusion of allogeneic blood products may be considered a pragmatic approach.

## Conclusion

A goal-directed coagulation algorithm including a target haematocrit-range including frequent and repeated haematocrit measurement may lead to less transfusion of RBC compared to only a lower haematocrit limit, when treating severely traumatized patients.

## Additional file

Additional file 1: Transfusion and coagulation algorithm of the Cantonal Hospital Lucerne (LUKS-Alg). (PDF 23 kb)

## Abbreviations

ABGA: Arterial blood gas analysis
aPTT: Activated partial thromboplastin time
BE: Base excess
CI: Confidence interval
ED: Emergency department
FFP: Fresh frozen plasma
GCS: Glasgow coma scale
ICU: Intensive care unit
INR: International normalized ratio
ISS: Injury severity score
LUKS: Cantonal Hospital Lucerne
LUKS-Alg: Transfusion and coagulation algorithm of the Cantonal Hospital Lucerne
OR: Odds ratio
PCC: Prothrombin complex concentrate
PLT: Platelet concentrate
RBC: Red Blood Cell concentrate
ROTEM: Rotational Thomboelastometry
SD: Standard deviation
TXA: Tranexamic acid
USZ: University Hospital Zurich
USZ-Alg: Transfusion and coagulation algorithm of the University Hospital Zurich
