# Supplementary material for: Comparison of two different coagulation algorithms on the use of allogenic blood products and coagulation factors in severely injured trauma patients: a retrospective, multicentre, observational study
Source: Scand J Trauma Resusc Emerg Med. 2018 Jan 8;26:4. doi: 10.1186/s13049-017-0463-0 (PMC5759800; doi:10.1186/s13049-017-0463-0)
Supplement: Additional file 1: — Transfusion and coagulation algorithm of the Cantonal Hospital Lucerne (LUKS-Alg). (PDF 23 kb) [file 13049_2017_463_MOESM1_ESM.pdf]

# Goal-directed coagulation and transfusion algorithm oft the Institute of Anesthesiology, Kantonsspital Lucerne

## Basic conditions:

Volume: Fluid therapy with balanced crystalloid solution (balanced Gelatin solution may be considered)

Always: Temperature ≥ 35°C + pH ≥ 7.2 + Ionized Calcium ≥ 1.15mmol/L + Hematocrit ≥ 0.21

Patient history: something known about medication or coagulation disorders? Consider Desmopressin 0.3µg/kg in case of platelet-inhibitors intake. Antagonize heparin with protamine 1:1.

## Diffuse microvascular bleeding (> 50% Bloodvolume):

Extem spindle-shaped + Aptem normal: Hyperfibrinolysis → 2g Tranexamic acid (15-30mg/kg)

Fibtem-MCF ≤ 7mm: 1-2g Fibrinogen (max. 3x2g, after that factor XIII 15U/kg); Target value: MCF ≥ 7mm (fibrinogen ≥1.5-2.0 g/L)

## Laboratory:

Hematocrit, platelet count, prothrombin time, INR, activated partial thromboplastin time, fibrinogen concentration, Rotem (Extem, Intem, Fibtem, Aptem), factor XIII, Anti-Xa, thrombin time

## Ongoing bleeding (5-10 Liters)

Extem/Intem: CT prolonged (Extem >80s, Intem >240s): Prothrombin complex concentrate 500-2'000U (according to bodyweight and INR)

Extem/Intem: CT normal (Extem <80s, Intem <240s) + MCF <40mm: Factor XIII 1250U (15U/kg) if Fibtem-MCF stays ≤ 7mm despite fibrinogen administration. Target value factor XIII ≥ 60% (if ≤ 40% → 20U/kg / if ≤ 50% → 15U/kg). Further administration of fibrinogen, as long as Fibtem-MCF ≤ 7mm

Extem/Intem: MCF < 40mm although Fibtem-MCF >10mm + Tc < 50'000/mm³: 1 Platelet concentrate

## Other potential measures:

Arterial ligation, cloths compression, aorta clamping, embolization, hysterectomy

## Further ongoing bleeding (>10 Liters)

FFP: 15ml/kg (~ 4U) → for faktors XI and V

If prognosis isn't infaust and temperature, pH, platelet count and fibrinogen target-values are achieved: rFVIIa: 90µg/kg (≈ 7.2mg) – once repeatable after 30min.

If further ongoing diffuse bleeding, contemplate von Wilebrand factor concentrate 2000U (20-30U/kg factor VIII)

**Trauma patient (age <50yrs + not infaust prognosis + Temperature >35°C + diffuse bleeding) or uterine atony** (Extem-MCF <25mm + Fibtem-MCF <4mm + Extem-CT >90s or at the extreme, possible to give without laboratory):

2g Tranexamic acid + 4g Fibrinogen + 1'000U Prothrombin complex concentrate + 4 erythrocytes concentrates (blood group 0 negative) + 1250U factor XIII
